# Glycoengineered anti-CD39 promotes anticancer responses by depleting suppressive cells and inhibiting angiogenesis in tumor models

**DOI:** 10.1172/JCI157431

**Published:** 2022-07-01

**Authors:** Haohai Zhang, Lili Feng, Paola de Andrade Mello, Changchuin Mao, Richard Near, Eva Csizmadia, Leo Li-Ying Chan, Keiichi Enjyoji, Wenda Gao, Haitao Zhao, Simon C. Robson

**Affiliations:** 1 Center for Inflammation Research, Department of Anesthesia, Critical Care & Pain Medicine, Beth Israel Deaconess Medical Center, Harvard Medical School, Boston, Massachusetts, USA.; 2 Department of Hematology, Shandong Provincial Hospital Affiliated to Shandong First Medical University, Jinan, China.; 3 Antagen Institute for Biomedical Research, Boston, Massachusetts, USA.; 4 Department of Advanced Technology R&D, Nexcelom from PerkinElmer, Lawrence, Massachusetts, USA.; 5 Department of Medicine, Division of Gastroenterology, Beth Israel Deaconess Medical Center, Harvard Medical School, Boston, Massachusetts, USA.; 6 Department of Liver Surgery, State Key Laboratory of Complex Severe and Rare Diseases, Peking Union Medical College Hospital, Chinese Academy of Medical Sciences and Peking Union Medical College (CAMS & PUMC), Beijing, China.

**Keywords:** Angiogenesis, Immunology, Cancer immunotherapy

## Abstract

Immunosuppressive cells accumulating in the tumor microenvironment constitute a formidable barrier that interferes with current immunotherapeutic approaches. A unifying feature of these tumor-associated immune and vascular endothelial cells appears to be the elevated expression of ectonucleotidase CD39, which in tandem with ecto-5′-nucleotidase CD73, catalyzes the conversion of extracellular ATP into adenosine. We glycoengineered an afucosylated anti-CD39 IgG2c and tested this reagent in mouse melanoma and colorectal tumor models. We identified major biological effects of this approach on cancer growth, associated with depletion of immunosuppressive cells, mediated through enhanced Fcγ receptor–directed (FcγR-directed), antibody-dependent cellular cytotoxicity (ADCC). Furthermore, regulatory/exhausted T cells lost CD39 expression, as a consequence of antibody-mediated trogocytosis. Most strikingly, tumor-associated macrophages and endothelial cells with high CD39 expression were effectively depleted following antibody treatment, thereby blocking angiogenesis. Tumor site–specific cellular modulation and lack of angiogenesis synergized with chemotherapy and anti–PD-L1 immunotherapy in experimental tumor models. We conclude that depleting suppressive cells and targeting tumor vasculature, through administration of afucosylated anti-CD39 antibody and the activation of ADCC, comprises an improved, purinergic system–modulating strategy for cancer therapy.

## Graphical abstract

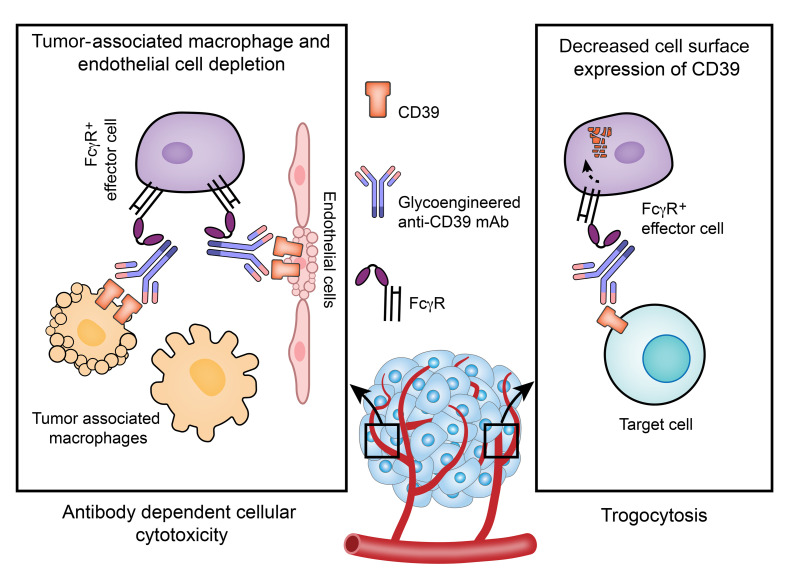

## Introduction

Cancer treatment has been radically transformed by immunotherapies that can unleash antitumor immune responses, as highlighted by the utility of checkpoint blockade antibodies against PD-1/PD-L1 and CTLA-4. These therapeutic antibodies have become a part of standard care for many types of cancer. However, the overall response rate for immune checkpoint inhibitor monotherapy is still in the range of 20%–40% (1), suggesting that additional immunosuppressive mechanisms exist in the tumor microenvironment (TME).

The TME is enriched with many types of immunosuppressive cells, such as regulatory T cells (Tregs), myeloid-derived suppressor cells (MDSCs), and tumor-associated macrophages (TAMs) (2). In addition to expressing ligands for inhibitory receptors on antitumor T cells, these cells favor tumor outgrowth through several other suppressive mechanisms. One common feature shared by most immunosuppressive cells is the upregulation of CD39, which is a rate-limiting ectonucleotidase controlling extracellular ATP (eATP) metabolism to produce nucleosides such as adenosine. We previously reported that Tregs coexpress CD39 and CD73, and generate adenosine as one of the major suppressive mechanisms (3). Subsequently, CD39 has been found to be a biomarker for MDSCs (4), TAMs (5), angiogenic tumor-associated endothelial cells (TAECs) (6), as well as exhausted CD8^+^ T cells (7). High-level expression of CD39 by immune cells and TAECs (8) in the TME results in elevation of the concentration of the highly immunosuppressive adenosine, estimated at 1000-fold more than in normal tissues (9).

One way to neutralize the effect of CD39 is to target the active site and hence possibly block most of the intrinsic ectonucleotidase activity. However, those anti-CD39 (αCD39) antibodies relying on the blockade of phosphohydrolytic enzymatic activities as the sole mechanism of action may suffer from the following disadvantages. First, it is difficult for antibodies to directly target the putative catalytic site of CD39 and completely block ectonucleotidase activity in vitro. Select CD39-neutralizing antibodies (TTX-030 and IPH5201) developed to primarily operate in this manner are in the early stages of clinical trials (10–12). TTX-030 inhibits ecto-enzymatic activity of soluble CD39 by approximately 55% in vitro (13). IPH5201 appears to have stronger inhibitory activity and maximally blocks around 70% of membrane-associated CD39 activity in cellular assays in vitro, albeit this requires high antibody concentrations, of the order of 10 μg/mL. Moreover, we note that the use of IPH5201 in a human CD39–knockin mouse did not significantly impact tumor growth alone. In this model, substantive anticancer effects required additive activities of oxaliplatin, a chemotherapeutic drug that induces ATP release (11). Secondly, CD39-neutralizing antibodies may not achieve the required concentrations to block ectonucleotidase activity because widespread, off-target expression, as in the vasculature of normal organs (8) and the poor penetration of antibodies into tumors (14, 15). Third, because of the existence of other ectoenzymes that catalyze eATP in tandem with CD73, suboptimal inhibition of persistent CD39 biochemical activity may not wholly prevent the accumulation of adenosine in the TME (16). This is particularly relevant given heightened ectonucleotidase functions, which can be linked to Treg apoptosis within tumors (17) and the general “hypoxic-adenosinergic environment” that induces immunosuppressive pathways, as well as driving angiogenesis (18–20). Considering these disadvantages of neutralizing checkpoint inhibitor–type mAbs against CD39, we sought to target this dominant ectonucleotidase using a bioengineering strategy to harness the cytotoxic potential of such biological reagents.

We proposed that deletion and/or removal of all CD39-expressing alive or apoptotic, immune and vascular endothelial cells from the TME would achieve profound antitumor outcomes. One way of inducing depletion is to use mAbs with high effector functions to target those immune or vascular cells of interest that express the antigen. Glycoengineering can be achieved by enzymatically altering antibody glycan substitution or by producing antibodies by cells genetically altered in enzymes involved in antibody glycosylation in vitro. Subtle differences in antibody glycan structure can greatly affect effector functions, such as antibody-dependent cellular cytotoxicity (ADCC) and antibody-dependent cellular phagocytosis (ADCP) (21). By further application of αCD39 class switching to bolster FcγR interactions in addition to such glycoengineering, we can substantially heighten effector functions in vitro (22).

In these current studies, the administration of a glycoengineered afucosylated (Afuc) αCD39 IgG2c mAb results in overall loss of CD39 expression within the TME, with effective depletion of CD39^hi^ cells (immunosuppressive TAMs and TAECs), which then unleashes immune responsiveness and blocks tumor angiogenesis. Monotherapy with Afuc αCD39 IgG2c elicits extensive tumor necrosis and growth inhibition, which is observed in both immunological “hot” colorectal MC38 and “cold” melanoma B16F10 tumors. Furthermore, we have demonstrated that Afuc αCD39 IgG2c considerably increases the antitumor efficacy of low-dose chemotherapy and PD-L1 blockade in these experimental models.

Importantly, systemic depletion of Tregs may result in deleterious autoimmunity. Hence it is crucially important to target what may be considered terminally differentiated effector Tregs within tumors, given specific expression of CD39 by these cells and recognition of tumor-associated antigens (23). Given this, the use of CD39 should be more precise than other modalities in targeting pathogenic effector Tregs within tumors. This benefit may be, at least in part, associated with the upregulation of this ectonucleotidase in the hypoxic-adenosinergic environment and allows for further combinational approaches, e.g., hyperoxia (18, 19, 24). Therefore, targeting CD39 with enhanced antibody effector functionality provides a broader anticancer strategy, allowing for greater efficacy and higher specificity, limiting systemic deletion of Tregs and decreasing risks for induced autoimmunity.

## Results

### High levels of CD39 expression on tumor-associated monocytes/TAMs and TAECs.

**Figure 1:**
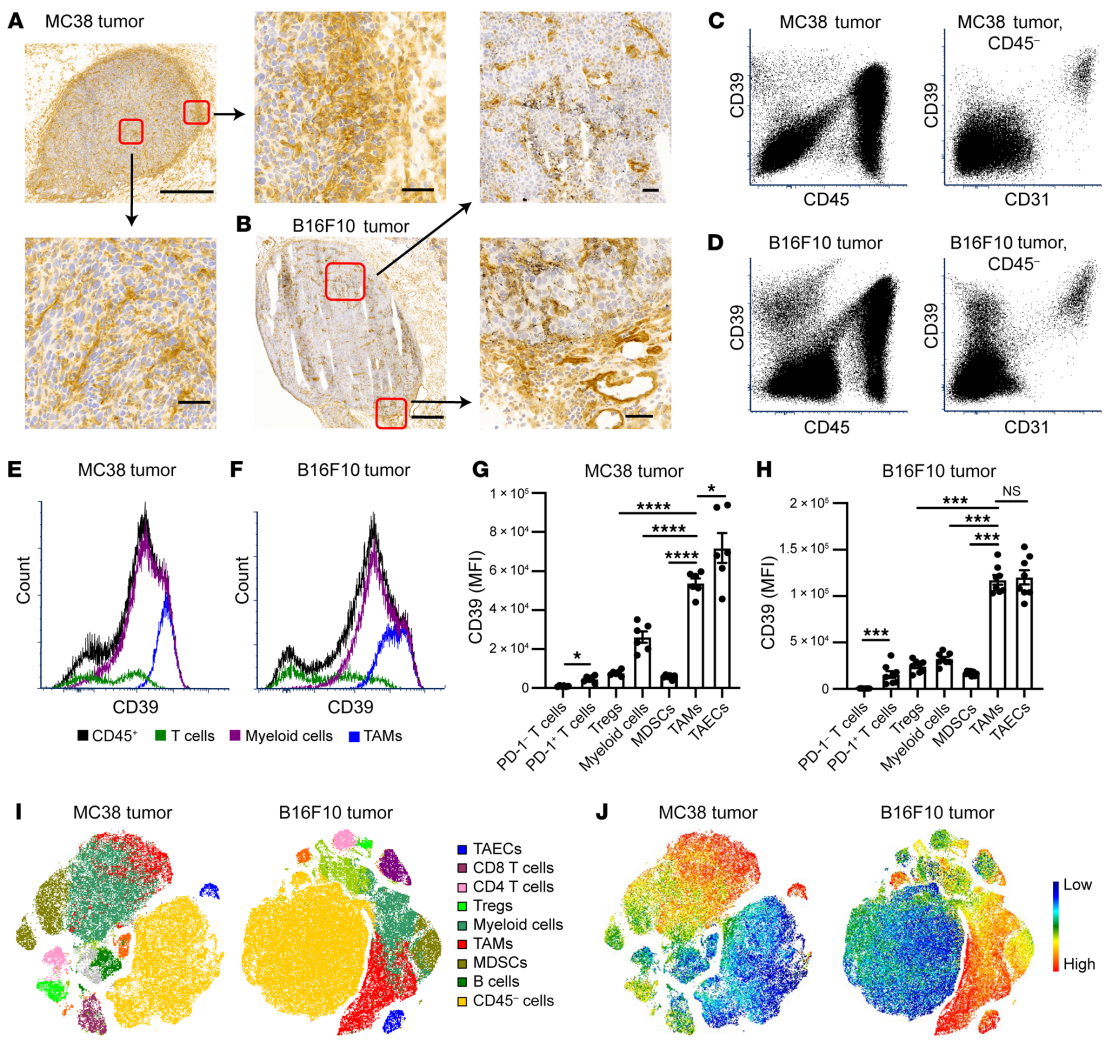
CD39 is differentially expressed on tumors cell subsets, and expression level peaks on tumor-associated endothelial cells (TAECs) and tumor-associated macrophages (TAMs). MC38 or B16F10 tumor cells were injected subcutaneously into WT C57BL/6 mice. Tissues were harvested on day 11 (MC38) and day 14 (B16F10) after tumor cell inoculation. (**A**) Representative images of CD39 immunohistochemistry (IHC) staining on MC38 tumor frozen sections. Scale bars: 500 μm and 50 μm (magnified views). (**B**) Representative images of CD39 IHC staining on B16F10 tumor frozen sections. Scale bars: 500 μm and 50 μm (magnified views). (**C**–**F**) CD39 expression on MC38 and B16F10 total tumor cells measured by flow cytometry. (**C** and **D**) Representative dot plots of CD39 expression on CD45^+^ and CD45^–^ populations (left plots). CD39 expression on CD45^–^ cells was further analyzed based on CD31 expression (right plots). (**E** and **F**) Representative histograms of CD39 expression on CD45^+^ population subsets, including T cells (CD3^+^), myeloid cells (CD3^–^CD11b^+^), and TAMs (CD3^–^CD11b^+^Gr-1^–^F4/80^hi^). (**G** and **H**) Quantification of CD39 median fluorescence intensity (MFI) on different cell subsets in MC38 (*n =* 6) (**G**) and B16F10 tumors (*n =* 7) (**H**). Data are shown as mean ± SEM. Repeated-measures 1-way ANOVA with Geisser-Greenhouse correction was used for statistical analysis. (**I** and **J**) Representative tSNE plots of the whole tumors showing the cell types and their respective CD39 expression. Data in **A**–**J** are representative of at least 2 independent experiments. *P < 0.05; ***P < 0.001; ****P < 0.0001. NS, not significant.

We first investigated CD39 expression patterns in syngeneic tumor models in wild-type (WT) C57BL/6 mice. Immunohistochemistry (IHC) revealed heterogeneous expression patterns of CD39 throughout MC38 tumors, with strong expression of CD39 among cells infiltrating the tumor and those accumulated at the tumor borders (Figure 1A). The most robust CD39 staining in central areas of tumors was on vascular endothelium (Figure 1A). When compared with MC38 tumors, the overall CD39 expression was less abundant in B16F10 tumors. In the latter case, the tumor mass was surrounded by a discontinuous layer of CD39^+^ cells (Figure 1B), with less dense CD39^+^ cell infiltrates (Figure 1B).

We next isolated and analyzed cell subsets in tumors for CD39 expression using multicolor flow cytometry. High-level CD39 expression was noted on CD45^+^ tumor-infiltrating immune cells (TICs) (Figure 1, C and D). When the CD45^–^ populations were further gated on CD31 expression, distinctive high CD39 expression was noted on TAECs (CD45^–^CD31^+^), whereas tumor cells (CD45^–^CD31^–^) per se did not express CD39 (Figure 1, C and D). Among the CD45^+^ TICs, CD3^–^CD11b^+^ myeloid cells expressed greater levels of CD39 and accounted for the vast majority of TICs, while the smaller population of CD3^+^CD11b^–^ T cells expressed lower levels of CD39 (Figure 1, E and F, and Supplemental Figure 1A; supplemental material available online with this article; https://doi.org/10.1172/JCI157431DS1). Within the CD11b^+^ tumor myeloid cell compartment, TAMs expressed the highest levels of CD39, second only to TAECs (Figure 1, E–H).

The median fluorescence intensities of CD39 on TAMs and TAECs were approximately 5- to 10-fold higher than those on PD-1^+^ T cells, Tregs, and MDSCs (Figure 1, G and H). Minimal CD39 expression was observed on these cells in the spleens from tumor-bearing mice, suggesting that the TME was associated with cellular CD39 upregulation (Supplemental Figure 1B). In addition, we found that when compared with TAECs, the majority of endothelial cells in the kidney, lung, and liver had lower levels of CD39 expression, whereas endothelial cells in the heart had a similar level of CD39 expression (Supplemental Figure 1C).

We further employed t-distributed stochastic neighbor embedding (tSNE) plots to develop single-cell CD39 expression landscapes in both MC38 and B16F10 tumors (Figure 1, I and J). The high-level expression of CD39 on TAMs and TAECs, critical contributors to tumor progression and chemotherapy resistance (25, 26), can be visualized in both tumor types (Figure 1, I and J).

### αCD39 antibody with enhanced effector function has marked antitumor activity in vivo.

**Figure 2:**
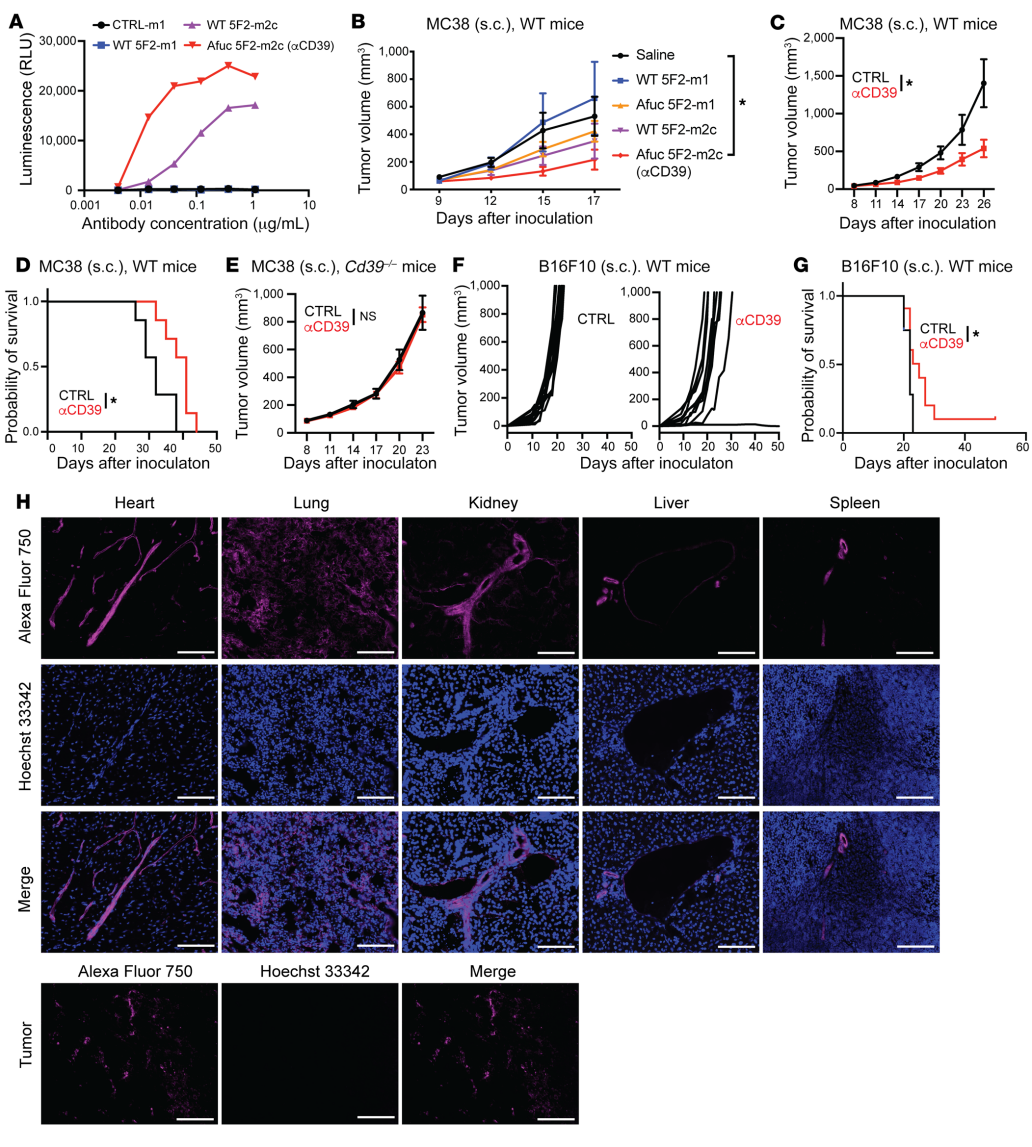
Afucosylated anti-mCD39 Ab (Afuc 5F2-m2c, αCD39) boosts ADCC function, inhibiting tumor growth in vivo. (**A**) CD39-overexpressing CHO cells were treated with antibodies for 30 minutes. Effector cells (Jurkat cells expressing mFcγRIV and NFAT-RE driving luciferase expression and luminescence signal) were added to the culture and incubated (6 hours). Afuc 5F2-m2c Ab exhibited the highest ADCC function in vitro. (**B**) MC38 tumor cells were engrafted in WT Foxp3EGFP–knockin reporter mice on day 0. Antibodies were administered (200 μg/mouse) intraperitoneally on days 9, 12, and 15. Saline (*n =* 7), WT 5F2-m1 (*n =* 4), Afuc 5F2-m1 (*n =* 4), WT 5F2-m2c (*n =* 5), Afuc 5F2-m2c (*n =* 4). (**C** and **E**) MC38 tumor cells were engrafted in WT mice (**C** and **D**) or *Cd39^–/–^* mice (**E**) (day 0). MC38 tumor–bearing mice received 5 mg/kg antibodies intraperitoneally (*n =* 7 per group) on days 8, 11, 14, and 17. Tumor growth (**C** and **E**) and/or time point when tumor size (2000 mm^3^) required euthanasia of mice (**D**) were measured. (**F** and **G**) Mice bearing subcutaneous B16F10 tumors received 10 mg/kg Ab treatment intraperitoneally on days 3, 6, and 9 after tumor inoculation. Tumor growth curve (**F**) and time to euthanasia (**G**) of mice in the control (CTRL) group (*n =* 12) and αCD39 mAb–treated group (*n =* 11) are presented. Data in **B**–**E** are shown as mean ± SEM. Two-way ANOVA (**B**, **C**, and **E**) and the log-rank test (**D** and **G**) were used for statistical analyses. (**H**) Mice bearing subcutaneous MC38 tumors received 5 mg/kg AF750-labeled αCD39 mAb (magenta) treatment intraperitoneally on day 11 after tumor inoculation (*n =* 4). Tissues were harvested on day 12. One hour before sacrifice, 15 mg/kg Hoechst 33342 (blue) was injected into tumor-bearing mice via tail vein. IHC images of representative fields of MC38 tumors and other organs. Scale bars: 100 μm. Data in **A**–**H** are representative of at least 2 independent experiments. *P < 0.05. NS, not significant.

Given the strong correlation between CD39 and the TME, we sought to test whether targeting CD39 could alter the suppressive nature of the TME and affect tumor growth. As recently documented, we engineered a non-neutralizing mouse IgG1 antibody against murine CD39 (clone 5F2) and screened an isotype-switched hybridoma subline of the mIgG2c isotype for more potent ADCC/ADCP activities (22). To further boost the effector functions, the fucosyltransferase 8 (*Fut8*) gene was deleted with CRISPR in the two 5F2 hybridomas to produce fully Afuc antibodies. Antibody isotype switching and genetic engineering of the hybridomas did not alter the binding affinity and specificity of the antibodies (Supplemental Figure 2, A–D), but dramatically increased ADCC activity (Figure 2A). This ADCC activity was measured by a luciferase reporter assay, where mouse CD39–expressing (mCD39-expressing) CHO cells serve as the target cells and mouse FcγRIV–expressing Jurkat cells serve as the surrogate effectors. In the Jurkat cells, the luciferase gene was driven by the nuclear factor of activated T cells (NFAT) response element upon the activation of FcγRIV in the presence of antibodies recognizing the target cells. When compared with WT 5F2-m2c, Afuc 5F2-m2c exhibited dramatically increased ADCC activity through both mFcγRIII and mFcγRIV, whereas afucosylation did not alter ADCC activity of 5F2-m1 through mFcγRIII or mFcγRIV (22), the latter of which does not bind mIgG1. Notably, none of these mAbs markedly inhibited CD39 ectonucleotidase activity in vitro (Supplemental Figure 2E).

With this 4-antibody-variant collection, we contrasted antitumor activities in established MC38 tumors. To decrease direct interactions of the antibodies with systemic vasculature, antibodies were administered via intraperitoneal injection. The results show that WT 5F2-m1 did not affect tumor growth, whereas Afuc 5F2-m2c had the strongest antitumor activity; Afuc 5F2-m1 and WT 5F2-m2c were intermediate in effects (Figure 2B). To generate a better control for Afuc 5F2-m2c, an irrelevant Afuc mIgG2c antibody (1D9) was produced by CRISPR-engineered *Fut8*-knockout hybridoma. This Afuc antibody (1D9) did not affect tumor growth (Supplemental Figure 2F). Compared with Afuc 1D9 (CTRL), Afuc 5F2-m2c (hereafter referred to as αCD39) significantly limited the tumor growth and prolonged time to euthanasia of MC38 tumor–bearing mice (Figure 2, C and D). This antitumor activity was not seen in MC38 tumor–bearing *Cd39^–/–^* mice, excluding possible off-target effects (Figure 2E). In addition to the MC38 tumor model, we also investigated the antitumor effects of αCD39 mAb in the subcutaneous B16F10 tumor model. When compared with the CTRL group, tumor growth in the αCD39 mAb–treated group was substantively delayed (Figure 2F), and time to euthanasia was significantly prolonged (Figure 2G).

To help understand the mechanism of action of the αCD39 mAb, we investigated the biodistribution of the antibody. The αCD39 mAb was first labeled with Alexa Fluor 750 (AF750), and the specificity of αCD39 mAb was not changed by AF750 labeling (Supplemental Figure 2G). Then, the AF750-labeled mAb was injected into MC38 tumor–bearing mice. Twenty-four hours later, we injected Hoechst 33342 into mice via the tail vein and harvested tumors and vital organs 1 hour later. The distribution of αCD39 mAb was then visualized by fluorescent IHC.

We found that the strongest AF750 signal was associated with vascular endothelium (Figure 2H). The larger field of view of tissues, as depicted in Figure 2H, is shown in Supplemental Figure 2H. The staining pattern of AF750 in tumors also appeared unique when compared with normal vasculature in heart, lung, and visceral organs. Most AF750 staining appeared in ring-like structures surrounding a necrotic tumor core, which Hoechst 33342 did not penetrate (Figure 2H and Supplemental Figure 2, H and I). These results prompted further study of the putative antiangiogenic, vascular targeting effects of the αCD39 mAb.

### αCD39 mAb selectively depletes TAECs and TAMs, blocking angiogenesis in vivo.

**Figure 3:**
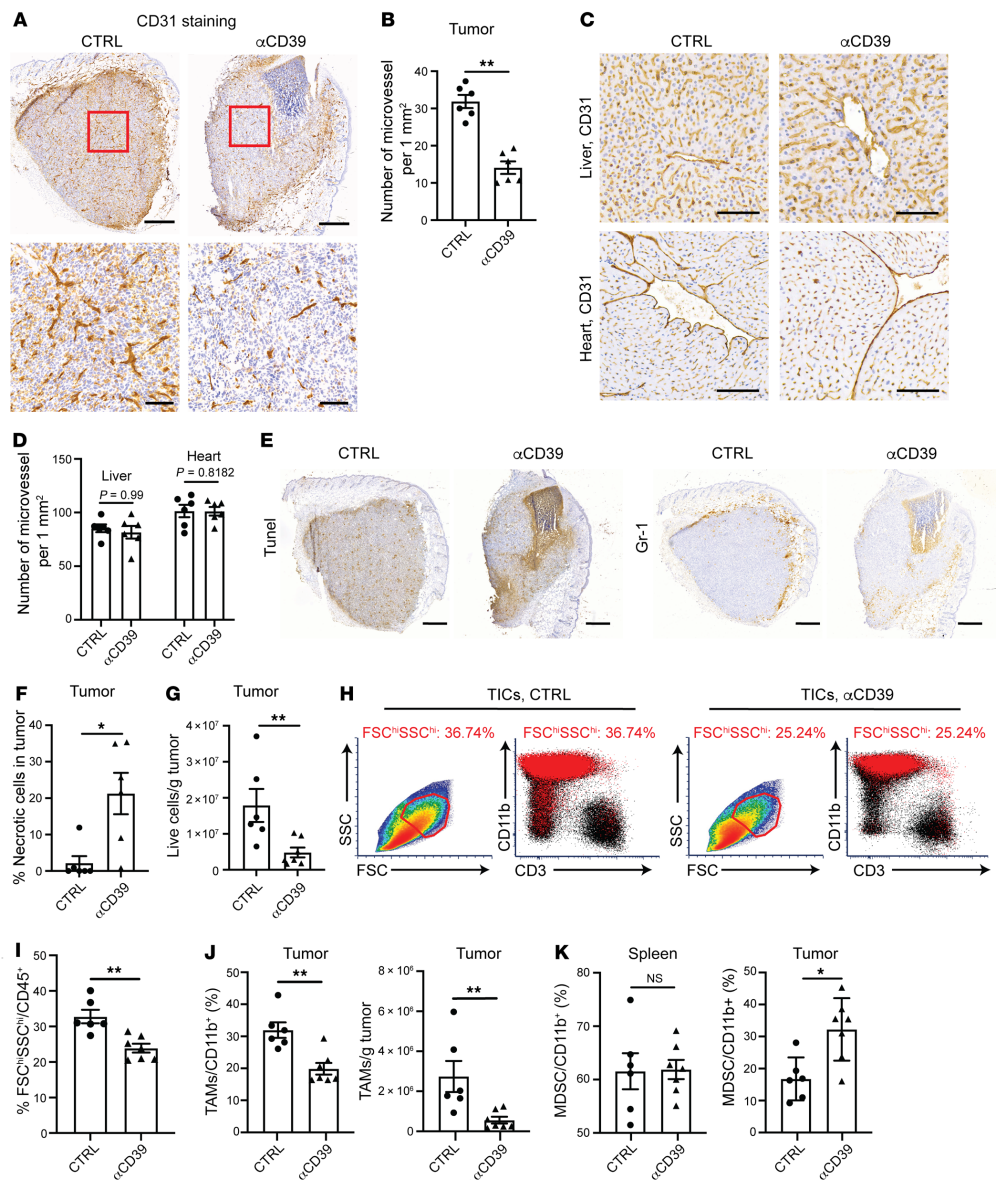
αCD39 mAb drives TAEC and TAM depletion in vivo. MC38 tumor cells were injected subcutaneously into WT C57BL/6 mice (day 0). Tumor tissues were collected on day 12 after being treated with 5 mg/kg Ab on days 8 and 11. (**A**) IHC images of representative MC38 tumors immunostained with anti-CD31. Scale bars: 500 μm (top) and 100 μm (bottom). (**B**) Quantification of microvessel density in MC38 tumor based on CD31 IHC staining. (**C**) Representative images of liver and heart from MC38 tumor–bearing mice stained with anti-CD31. Scale bars: 100 μm. (**D**) Quantification of microvessel density in liver and heart from MC38 tumor–bearing mice based on CD31 IHC staining. (**E**) Representative images of MC38 tumors immunostained with TUNEL and anti–Gr-1. Scale bars: 500 μm. (**F**) Quantitative estimates of the necrotic area in the tumor based on TUNEL staining. (**G**) Flow cytometry analysis of the absolute number of live cells in the tumor. (**H**) Representative FACS plots showing a reduction in CD45^+^ live cell population with high FSC and SSC after αCD39 treatment (right), consisting of mainly myeloid cells (CD3^–^CD11b^+^) (bottom right). (**I**) Percentage of FSC^hi^SSC^hi^ cells in the tumor-infiltrating immune cells (TICs). (**J**) Quantification of the cell ratio (left) and absolute cell number (right) of TAMs (CD11b^+^Gr-1^–^F4/80^hi^) within tumors. (**K**) Quantification of MDSC (CD11b^+^Gr-1^+^) cell ratio in spleen (left) and tumor (right). Data are shown as mean ± SEM. The Mann-Whitney test was used for the statistical analysis (**B**, **D**, **F**, **G**, and **I**–**K**). Data in **A**–**K** are representative of at least 2 independent experiments. *P < 0.05; **P < 0.01. NS, not significant.

By IgG subclass switching and glycoengineering, we transformed an αCD39 mAb to exhibit strong effector functions. As TAECs and TAMs express high levels of CD39, we next investigated whether the αCD39 mAb inhibited tumor growth via depletion of CD39^hi^ TAECs and TAMs. In the MC38 tumor model, we harvested tumor tissues 1 day after the second dose of antibody treatment for IHC or flow cytometry. CD31^+^ microvessels were densely formed throughout the tumor in the CTRL group. In contrast, tumor microvessels in the αCD39-treated group did not grow deep inside the tumor (Figure 3A).

CD39 is expressed on the normal, quiescent endothelium. While αCD39 mAb treatment substantially decreased the density of intratumoral microvessels (Figure 3B), CD31 IHC staining in livers and hearts from αCD39-treated mice did not reveal any differences in microvessel formation (Figure 3C) and CD31^+^ microvessel density (Figure 3D). To further address safety concerns, we injected the αCD39 mAb into tumor-free, healthy WT C57BL/6 mice. The treatments of αCD39 mAb at 100 μg, 200 μg, or 1000 μg were all well tolerated, demonstrating a favorable safety profile. There were no signs of hepatic or renal toxicity (as determined by blood chemistry) and no evidence for systemic thromboembolism or vascular injury (as assessed by histopathology) (Supplemental Table 1 and Supplemental Figure 3A). These data suggested that αCD39 mAb–mediated effects were largely tumor selective.

In addition, TUNEL staining demonstrated that αCD39 treatment resulted in increased tumor apoptotic/necrotic areas (Figure 3, E and F), closely overlapped with infiltrating Gr-1^+^ monocytes/macrophages (Figure 3E). The numbers of viable cells in tumors were shown by flow cytometry to be significantly decreased after αCD39 treatment, which is in line with the TUNEL staining result (Figure 3G). These data suggest that αCD39 targets TAECs, largely inhibiting pathological angiogenesis in tumors.

We next characterized the influence of αCD39 on CD45^+^ TICs by flow cytometry. αCD39 treatment decreased a subset of TICs with high forward and side scatter (FSC^hi^SSC^hi^ TICs), which were mainly CD3^–^CD11b^+^ myeloid cells (Figure 3, H and I). Within the myeloid population, the frequency and total number of TAMs (CD11b^+^Gr-1^–^F4/80^hi^) were decreased by αCD39 (Figure 3J), while those of MDSCs (CD11b^+^Gr-1^+^) were curiously increased (Figure 3K), possibly through chemoattraction by dying cells (27). In contrast, in the spleen compartment, the myeloid population was not significantly affected by αCD39 treatment (Figure 3K), again supporting the notion that αCD39-mediated effects were tumor selective and strongly correlated with CD39 expression levels. Interestingly, αCD39 reduced the total number of T cells isolated from the tumor, but the ratios of CD8^+^/Treg and CD4^+^ Treg/Teff were largely not changed (Supplemental Figure 3, B–E), suggesting that depleting CD39^+^ TAECs and TAMs acts as a dominant mechanism.

### αCD39 mAb mediates trogocytosis of surface CD39 in vitro and in vivo.

**Figure 4:**
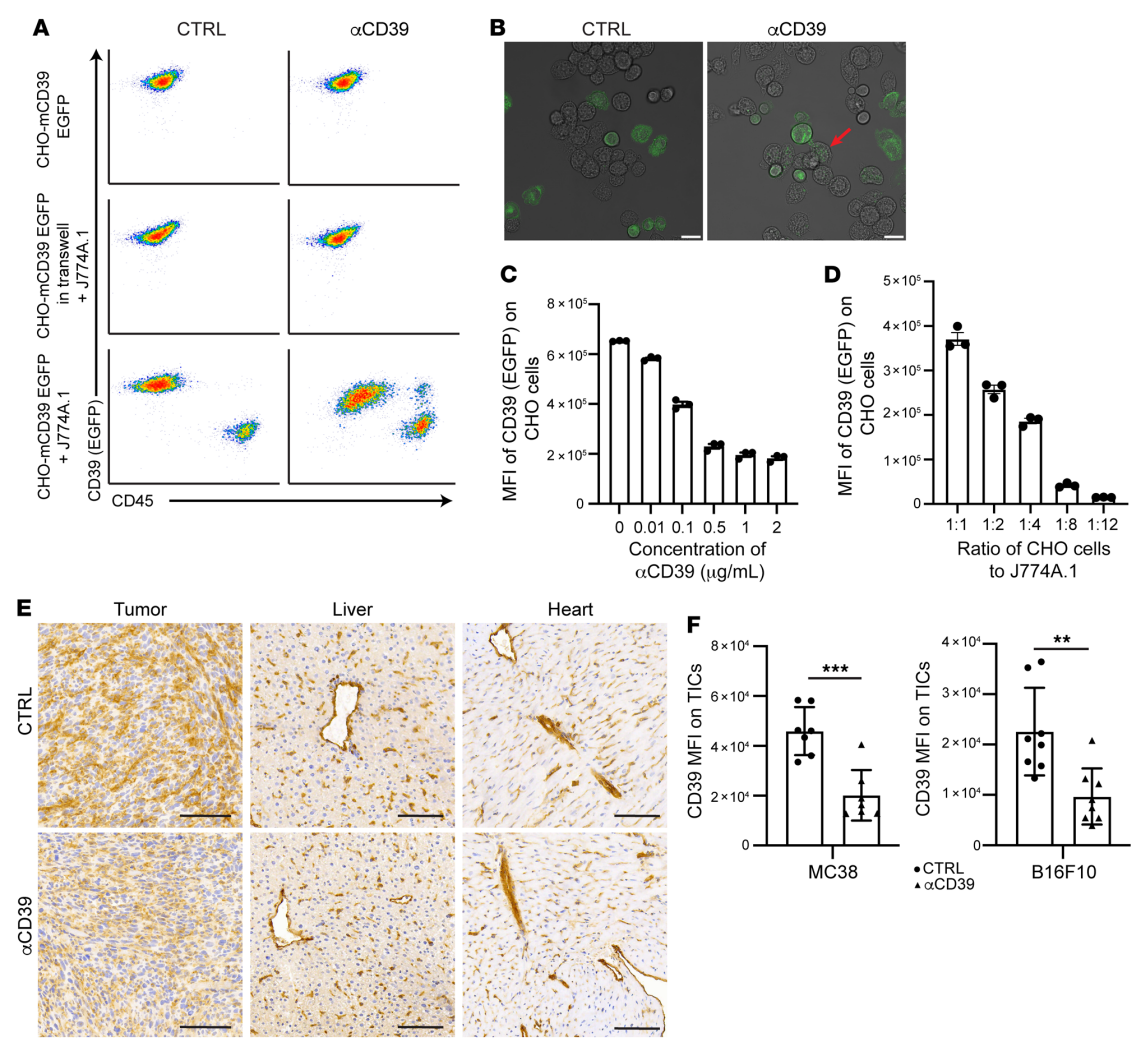
αCD39 mAb induces trogocytosis in vitro and in vivo. CHO cells overexpressing mCD39 tagged with EGFP were used as donor cells, while murine macrophage J774A.1 cells were used as acceptor cells in the trogocytosis assay. (**A**) CHO-mCD39-EGFP cells were cultured alone or in combination with J774A.1 cells. When CHO-mCD39 EGFP cells were cultured in Transwells, J774A.1 cells were positioned in the bottom chamber. The cocultures were treated with the indicated mAb for 48 hours. Flow cytometry analyses of signals on CHO-mCD39-EGFP cells in those cultures are shown. (**B**) Representative confocal microscopy images of cocultured CHO-mCD39-EGFP and J774A.1 cells treated with the indicated mAb. The red arrow marks the engulfed CD39-EGFP in J774A.1 cells. Scale bars: 20 μm. (**C**) Flow cytometry analysis of EGFP signal on CHO-mCD39-EGFP cells of cocultures treated with indicated concentrations of αCD39 mAb. (**D**) CHO-mCD39-EGFP and J774A.1 cells were cocultured at indicated ratios and treated with 2 μg/mL αCD39 mAb. Flow cytometry analysis of the EGFP signal on CHO-mCD39-EGFP cells is shown. (**E** and **F**) MC38 tumor–bearing mice were treated with 2 doses of 5 mg/kg control (CTRL) or αCD39 mAb on days 8 and 11 after tumor implantation. B16F10 tumor–bearing mice were treated with 2 doses of 10 mg/kg CTRL or αCD39 mAb on days 11 and 14 after tumor implantation. Tissues were harvested 24 hours after the second dose of treatment and processed for CD39 IHC staining (**E**) or FACS analysis (**F**). Scale bars: 100 μm. Data are shown as mean ± SEM. The Mann-Whitney test was used for the statistical analysis. Data in **A**–**F** are representative of at least 2 independent experiments. **P < 0.01, ***P < 0.001.

One major side effect of therapeutic antibody treatment in a clinical setting is the onset of cytokine release syndrome (CRS), which is in part induced by cytotoxicity toward targets (28). We next investigated whether this αCD39 mAb could induce trogocytosis to decrease cell surface CD39 expression but preserve cell viability (29). Murine monocyte-macrophage cell lines (J774A.1, acceptor cells) and CHO cells overexpressing mCD39 tagged with fluorescent protein EGFP (CHO-mCD39 EGFP, target cells) were cocultured. When target cells were incubated with αCD39 mAb in the absence of acceptor cells, the CD39 expression on CHO-mCD39-EGFP cells was unchanged (Figure 4A). When acceptor cells were included in the coculture system, αCD39 mAb markedly decreased CD39 levels on target cells (Figure 4A). This process requires cell-cell contact between acceptor cells and target cells, as when they were separated by Transwells no CD39 reduction was observed (Figure 4A).

To gain further insight into the mechanism of αCD39-induced trogocytosis, live-cell imaging was performed using confocal microscopy to visualize the transfer of CD39 between different types of cells. We observed that when αCD39 mAb was present, J774A.1 physically interacted with the target cells and engulfed membrane portions containing CD39 from the target cells (Figure 4B and Supplemental Video 1). Factors affecting the extent of CD39 loss included the concentration of αCD39 mAb (Figure 4C), the ratios of target versus acceptor cells (Figure 4D), the isotype of antibody, and the type of acceptor cells (Supplemental Figure 4A). In addition, trogocytosis of CD39 was abrogated by preincubating cocultures with blocking antibodies against FcγR (FcγRIV, CD16.2; FcγRIII/II, CD16/32) before adding αCD39 mAb (Supplemental Figure 4B). These results strongly suggest that αCD39-mediated trogocytosis involves antibody Fc engagement with FcγR.

To examine whether αCD39-induced trogocytosis also occurred in vivo, we quantified CD39 expression in MC38 tumors by IHC and flow cytometry, after 2 doses of antibody treatment. The tissues of the tumors, host liver, and heart were stained with a polyclonal antibody against mCD39. There was substantial CD39 reduction on microvessels and other stroma cells of the tumor following αCD39 treatment, with no significant changes on the vasculature of the liver and heart (Figure 4E). We also performed FACS to investigate CD39 reduction on TICs, using a different αCD39 antibody (clone Duha59) with an epitope not overlapping with that of 5F2 as detecting antibody (Supplemental Figure 4C). Administration of αCD39 resulted in significant decreases in CD39 expression on TICs from both MC38 and B16F10 tumors (Figure 4F and Supplemental Figure 4D).

Based on these findings, a mechanism for antibody-triggered trogocytosis of surface CD39 is proposed: CD39^hi^ cells are opsonized with αCD39 mAb, which then engages FcγR on myeloid cells. This process brings monocytes/macrophages in close proximity to antibody-opsonized target cells to then exert ADCC and/or proceed to trogocytosis. This interaction ultimately results in either the elimination of CD39^hi^ cells or alternatively decreases levels of CD39 expression on target cells.

### αCD39 synergizes with suboptimal chemotherapy and immunotherapy.

**Figure 5:**
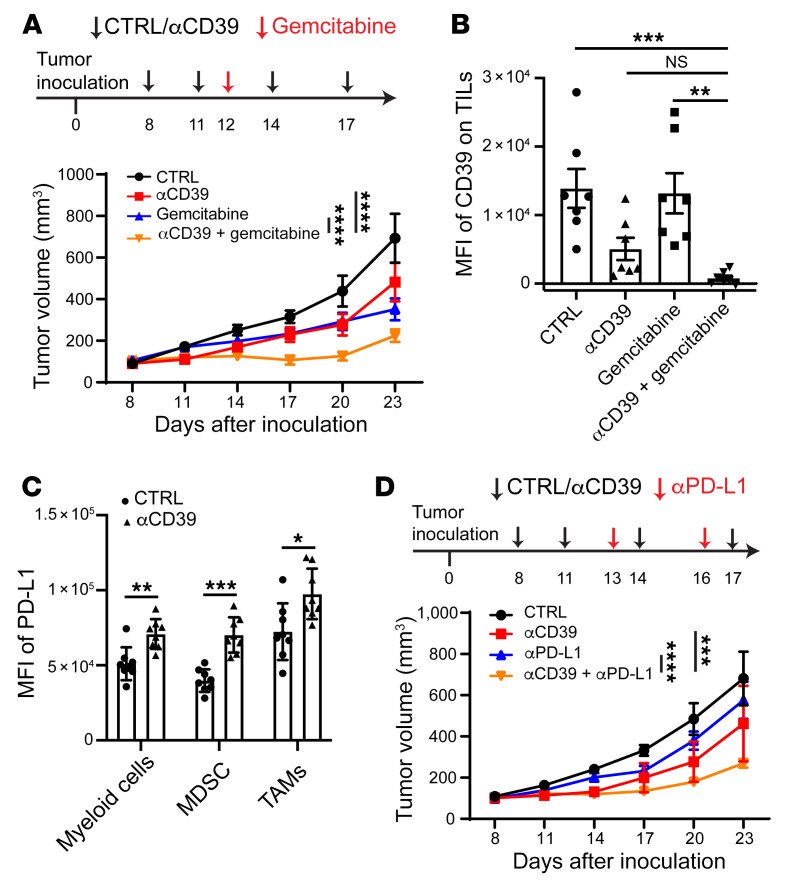
αCD39 mAb potentiates tumor sensitivity to chemotherapy and immune checkpoint therapy. (**A**) Treatment scheme (top) and MC38 tumor growth profile (bottom) after treatment with the indicated therapies. Control (CTRL) (*n =* 7), αCD39 (*n =* 8), gemcitabine (*n =* 7), αCD39 plus gemcitabine (*n =* 7). Data are shown as mean ± SEM. Two-way ANOVA was used for statistical analysis. (**B**) CD39 expression (MFI) on tumor-infiltrating T cells (TILs) analyzed by FACS. *n =* 7 mice in each group. Data are shown as mean ± SEM. Kruskal-Walis with post hoc Dunnett’s multiple-comparison test was used for the statistical analysis. (**C**) MC38 tumor–bearing mice were treated with 2 doses of αCD39 mAb on days 8 and 11 after tumor implantation. Tumors were harvested on day 12 and processed for FACS analysis. Quantification of PD-L1 expression on myeloid cells subsets in the tumor after treatment with αCD39 mAb is shown. *n =* 8 mice in each group. Data are shown as mean ± SEM. The Mann-Whitney test was used for the statistical analysis. (**D**) MC38 tumor growth curves in mice treated with the indicated antibodies. *n =* 7 mice in each group. Data are shown as mean ± SEM. Two-way ANOVA was used for statistical analysis. Data in **A**–**D** are representative of at least 2 independent experiments. *P < 0.05; **P < 0.01; ***P < 0.001; ****P < 0.0001. NS, not significant.

Efficient depletion of TAMs and/or TAECs through ADCC/ADCP and overall reduction of intratumoral CD39 content through trogocytosis were achieved by glycoengineered αCD39 mAb. Then, we hypothesized that this treatment might synergize with suboptimal chemotherapy and other avenues of immunotherapy. To test this, we established a combination regimen by treating tumor-bearing mice with 2 doses of αCD39 mAb prior to administration of gemcitabine at suboptimal doses and subsequently delivered 2 additional αCD39 doses. Indeed, the combination group exhibited marked tumor growth inhibition, in contrast to either the gemcitabine or αCD39 monotherapy group (Figure 5A). In the mouse system, CD39 marks Tregs (3, 30) or exhausted CD8^+^ T cells (7). Antibody treatment markedly diminished CD39 expression on tumor-infiltrating T cells, when used alone or in combination with gemcitabine (Figure 5B).

Upon αCD39 mAb treatment, MDSCs were recruited and these cells infiltrated apoptotic/necrotic areas of tumors (Figure 3E). Flow cytometry further demonstrated that MDSCs and TAMs had elevated levels of PD-L1 expression, which could be a compensatory mechanism for tumors to evade αCD39-boosted immune responses (Figure 5C). We investigated whether αCD39 mAb and PD-L1 blockade could synergize to enhance antitumor responses. When MC38 tumor–bearing mice were treated with αCD39 and αPD-L1 in combination, potent synergistic antitumor effects for more effective control of tumor growth than either the single agents were observed (Figure 5D). In addition to sensitizing tumors for suboptimal chemotherapy (or revoking chemoresistance), αCD39 treatment also synergizes with immunotherapy, such as anti–PD-L1 checkpoint blockade.

## Discussion

Purinergic signaling is known to have major impacts in inflammation and immunity (31). Importantly, eATP and adenosine have opposing effects on immune responses. While eATP serves as a danger molecule and can stimulate immune responses by activating P2 receptors, adenosine strongly suppresses immune responses by binding to ADORA2A receptors on immune cells (31, 32). Adenosinergic effects appear dominant in untreated cancer within the TME because of the high levels of ectonucleotidases that catalyze the formation of this nucleoside, the induction of ADORA receptors, TGF-β, and associated HIF1α signaling responses driven by hypoxia (19, 20, 24, 33).

We have shown that genetic ablation or pharmacological targeting of CD39, i.e., by blocking the conversion of eATP into adenosine, inhibits the growth of melanoma metastases (34), whereas overexpression of CD39 accelerates the growth of colon cancer in experimental models (35). Therefore, targeting CD39 with therapeutic antibodies to enhance eATP signaling over that of adenosinergic effects is a promising approach for cancer treatment, with theoretical advantages over just targeting CD73 or adenosinergic A2A receptors (32).

In these current studies, we have found high levels of CD39 expression within tumors, which means any neutralizing mAb will have to be dosed at high levels to block the associated ectonucleotidase activity. Additionally, high-level dosing of neutralizing αCD39 mAb could be associated with greater risk of systemic, off-target side-effects. Hence, we bioengineered non-neutralizing mAbs against CD39 and designed these to bolster targeting of regulatory lymphoid or myeloid immune cells, as well as the tumor vasculature. Unlike most existing CD39-neutralizing mAbs that minimize possible Fc interactions (36), we enhanced these effector functions of non-neutralizing αCD39 mAb by class switching and afucosylation. We noted that the antitumor activities are strongly boosted by this change in effector functionality, i.e., the antibody that harnesses ADCC outperforms other forms and is effective as monotherapy (Figure 2, A, B, and F).

In addition, after treatment with Afuc αCD39-m2c, differential levels of cellular expression of CD39 on various cell types in the TME predicted cell fate and therapeutic outcomes. Immune and vascular endothelial cells with the strongest CD39 expression (e.g., TAMs and TAECs) were effectively depleted from tumors (Figure 3). In comparison, cells with less CD39 expression (e.g., Tregs) were stripped of this ectonucleotidase via trogocytosis (Figure 3, Supplemental Figure 3, and Supplemental Video 1), a process that shaves antigens from membranes of antibody-opsonized cells (29). This mechanism decreases the overall CD39 content in the TME, leading to growth inhibition of both immunologically cold and hot tumors. This application of CD39 targeting also potentiates chemotherapy as well as other immunotherapeutic approaches and could be combined with strategies to target ADORA, as well as HIF1α (Figure 5 and refs. 24, 37).

The removal of the core fucose from N-glycans on Fc domains, to result in antibody afucosylation, can improve the binding of an antibody to activating FcγRs, leading to a 50- to 100-fold increase in ADCC activities (38–40). This approach has been used in other instances of targeting membrane glycoproteins as well as ecto-enzymes. For example, rituximab (αCD20), trastuzumab (αHER2/neu), and daratumumab (αCD38) are designed to trigger ADCC, but also decrease target expression levels via trogocytosis (41–43).

Trogocytosis has been demonstrated to be a key antitumor mechanism and involves the transfer of cell surface molecules from donor cells to acceptor cells (44). In this study, live-cell imaging demonstrated that antibody-mediated trogocytosis actively transferred donor CD39 molecules, tagged with EGFP, into the cytosol of acceptor macrophage cells. This process appeared to result in degradation, rather than display on the cell surface (Supplemental Video 1). As a result, total CD39 measurements in the TME were greatly decreased after treatment with these antibodies (Figure 4E). FcγR-mediated endocytosis might differentiate this process from other forms of trogocytosis that proceed between plasma membranes of different immune cells (45).

Clearly, Fab-dependent inhibition of CD39 ecto-enzymatic activity versus Fc-dependent depletion of CD39^+^ cells and/or decreasing surface expression represent 2 quite different strategies to target immunosuppressive adenosine generation within the TME. Both avenues have been explored in cancer treatment with biological therapeutics and may overlap. Indeed, antibodies against CTLA-4, GITR, ICOS, and OX40 were initially thought to solely act through the Fab portions to modulate effector T (Teff) cell responses. However, preclinical models indicated that these antibodies preferentially depleted Tregs in the tumor site, increasing the CD8^+^ to Treg ratio and promoting tumor rejection. This process is dependent on the presence of cells with activating FcγRs and of heightened immunoregulatory receptors on Tregs (46–48). In addition, anti–CTLA-4–triggered adverse events can be segregated from cancer immunotherapeutic effects. The former adverse effects are linked to Fab-mediated blockade of B7/CTLA-4 inhibitory signaling that turns off autoreactive T cells, whereas the latter beneficial outcome is achieved by FcγR-dependent depletion of Tregs in the TME (49–51).

Translating preclinical data into human studies requires caution, as Fc-FcγR interactions differ between these 2 systems. Ipilimumab, a human IgG1 mAb against CTLA-4, can trigger strong ADCC/ADCP by human (h)IgG1 Fc. In contrast, another CTLA-4 antibody, tremelimumab, which has the hIgG2 isotype, has fewer effector functions. Of note, ipilimumab has been approved for treating advanced melanoma (52), non–small cell lung cancer (53), and other indications (54), whereas tremelimumab has failed at least 6 clinical trials (55). Despite ipilimumab’s potentially depleting isotype, the contribution of ADCC and the role of FcγRs in dictating clinical activity of ipilimumab remains somewhat unclear (56, 57). Amivantamab, an αEGFR and αcMet low-fucose bispecific antibody, induces EGFR/cMet receptor downmodulation and antitumor activity via monocyte/macrophage trogocytosis (58). Other antibodies targeting stimulatory or inhibitory coreceptors, including OX40, LAG-3, CD70, and GITR, are being tested in cancer studies as Afuc forms to enhance antibody immunomodulatory functions in vivo (59).

The antitumor effects of the Afuc αCD39 antibody could be related to Treg depletion from the TME or to changes in ectonucleotidase expression on these cells. Importantly, abrogation of adenosinergic suppression of Teffs has been linked to promotion of T helper type 1 (Th1) responses and increased secretion of IFN-γ (3, 60). Higher IFN-γ levels have major effects on neovascularization, further resulting in antitumor effects (61). Remarkably, we did not observe meaningful changes in Teff/Treg ratios in the tumor when using Foxp3GFP indicator mice to track these (62). However, the levels of CD39 on Tregs in the TME were much lower than those on TAECs and TAMs (Figure 1, G and H), which may explain why Tregs were not substantially depleted. CD39 levels on nondepleted Tregs and other TICs were dramatically reduced via trogocytosis following treatment of Afuc αCD39 mAb as compared with control antibody (Supplemental Figure 4D).

Regardless of depletion of CD39^hi^ cells or stripping of CD39 molecules from the cell surface, the FcγR^+^ effector cells, e.g., macrophages and granulocytes, appear to be the important effectors of these processes. Macrophages are the major pathogenic cells mediating ADCC (63–65). In another model of ADCC and tumor cell targeting, limiting the number of macrophages decreased the therapeutic effect of the αCD30 antibody (SGN-30; ref. 65). MC38 tumors have higher immunogenicity and contain more macrophages. In contrast, B16F10 tumors have lower immunogenicity and are poorly infiltrated with macrophages. Although Simpson et al. demonstrated that an αCTLA-4 mAb depletes Tregs in B16F10 tumors (48), it should be noted that host animals also received an irradiated B16-BL6 tumor cell–based vaccine that secretes GM-CSF (GVAX) to boost macrophage infiltration.

Collectively, the extent of target cell depletion and target antigen trogocytosis by Afuc antibody positively correlates with the antigen expression levels on target cells and with the density of macrophages in the milieu. Hence, when high doses of Afuc αCD39 were administered into control or tumor-bearing mice, no obvious abnormalities were observed in the heart, liver, spleen, lung, and kidney tissues. More specifically, while TAEC depletion and tumor necrosis were specifically found in MC38 tumors, the endothelial cells in the liver and heart of healthy and tumor-bearing mice were not damaged.

In addition to the depletion of TAMs and TAECs, increases in tumor necrosis were observed after treatment with Afuc αCD39 mAb (Figure 3E). IHC showed that Gr-1^+^ MDSCs surrounded these necrotic areas in tumors. Sterile inflammation may attract inflammatory monocytes, which are CD11b^+^Gr-1^+^ and are responsible for debris clearance (27). Such CD11b^+^Gr-1^+^ cells have high levels of PD-L1 expression, which could explain the reason for the synergy between the Afuc αCD39 and αPD-L1 mAbs (Figure 5). This observation also suggests that Afuc bispecific antibodies targeting both CD39 and PD-L1 may provide further enhancement of antitumor activity.

Unlike the immunoregulatory receptors, where expression profiles are largely restricted to the immune cells, CD39 is more widely expressed and is present on the vascular endothelium (66, 67). We previously found that global CD39–knockout mice do not develop vasculopathy or obvious developmental abnormalities (8, 68). CD39 expression is substantially upregulated under pathological conditions, e.g., as in tumor angiogenesis (69, 70), where this is associated with hypoxia (19, 20, 24), stromal remodeling, and cell death (8, 68). In line with these features, we found that CD39 expression on tumor-infiltrating immune and vascular cells is markedly higher than in noncancerous tissues (Supplemental Figure 1B). The pivotal finding that Afuc αCD39 perturbed pathological angiogenesis in tumors but did not alter the normal vasculature is highly encouraging (Figure 3, A–D). Therefore, the Afuc αCD39 mAb appears to have a good safety profile, but further clinical studies are required.

In conclusion, we report an innovative strategy to more effectively modulate purinergic signaling and that achieves favorable outcomes in experimental cancer models. Instead of solely neutralizing CD39 ecto-enzymatic activities, glycoengineered Afuc αCD39 IgG2c mAbs selectively deplete CD39^hi^ TAECs and TAMs, abrogate angiogenesis, and decrease overall CD39 levels in the tumor. This strategy appears safe and is highly efficacious when applied in experimental solid tumors. This intervention could be applied either as a single agent or used synergistically with other modalities in combinational therapies to target the hypoxic-adenosinergic TME (20, 33). It might be also envisioned that glycoengineering of other therapeutic antibodies against purinergic targets, as exemplified by the findings in this study, could lead to more powerful reagents for cancer treatment.

## Methods

### Animals.

Global knockout *Cd39^–/–^* mice and WT (Foxp3GFP-knockin, *Cd39^+/+^*) mice on the C57BL/6 background were bred at Beth Israel Deaconess Medical Center (BIDMC). WT C57BL/6 mice were purchased from the Jackson Laboratory. All mice were kept in a temperature-controlled room with alternating 12-hour dark-light cycles.

### Cell lines.

Syngeneic murine MC38 colon cancer cells were provided by Nicholas P. Restifo (National Cancer Institute). Luciferase-expressing B16/F10 (luc-B16/F10), a genetically modified C57BL/6 mouse melanoma cell line, was a gift from Takashi Murakami (Jichi Medical School, Tochigi, Japan). The mouse monocyte-macrophage cell line J774A.1 was purchased from ATCC. NFAT-luc^+^ mFcγRIV Jurkat cells were obtained from Promega. Stable CHO cell lines expressing the full-length mouse *Entpd1* (CD39, UNIPROT: P55772) alone or in fusion with EGFP at the C-terminus, *Entpd2* (CD39L1, UNIPROT: O55026), *Entpd3* (CD39L3, UNIPROT: Q8BFW6), and *Entpd8* (UNIPROT: Q8K0L2) were generated using the Toggle-In method (Antagen). The genes were PCR cloned from the mouse splenocyte cDNA library. All the genes were cloned into the pTOG3 vector (Antagen) and the inserts were confirmed by sequencing (Genewiz). One microgram of each pTOG3 construct was cotransfected with 20 ng Cre-encoding pOG231 plasmid (Addgene) into CHO-E1 cells (Antagen) at a transcriptional hot spot via Cre-LoxP recombination–mediated cassette exchange, followed by hygromycin B selection (800 μg/mL) for 10 days. Single CHO clones were picked and confirmed by RT-PCR and FACS after staining with antibodies. All the clones within each line were isogenic with the same genomic integration by the Toggle-In method. All cell lines were maintained in culture flasks at 37°C in a 5% CO_2_ atmosphere at 100% humidity, except for NFAT-luc^+^ mFcγRIV Jurkat cells, which were thawed in a water bath at 37°C prior to use for experiments.

### αCD39 mAbs with different isotypes/classes and glycosylation.

Mouse mAb against mCD39 was previously generated by using cDNA immunization and an otherwise standard hybridoma approach (71). Briefly, *Cd39^–/–^* mice were immunized with pcDNA3.1 encoding mCD39, mCD39-overexpressing CHO cells, and recombinant mCD39 protein. Positive hybridoma clones were identified by FACS screening of antibody binding to GFP^+^ Tregs from Foxp3GFP-knockin mice. Among these, a clonal hybridoma cell line (5F2, mIgG1κ, WT 5F2-m1) was established by 96-well subcloning.

Further screening of 5F2 hybridoma subclones using ELISA and isotyping rapid test (Antagen) yielded a spontaneously switched isoform of the IgG2c isotype (WT 5F2-m2c), which is associated with heightened ADCC (72). RT-PCR of heavy and light chain genes and DNA sequencing confirmed that this isoform had identical sequences for Fab fragments but distinct sequences for Fc regions corresponding to mIgG1 and mIgG2c (data not shown).

Oligonucleotides for guide RNAs targeting the murine *Fut8* gene were cloned into sgRNA/Cas9n expression vector pX335 (Addgene), and electroporated into the 2 hybridoma cell lines. After electroporation, cells were cultured in DMEM with 10% FBS for 8 days before being stained with biotinylated *Lens culinaris* agglutinin (LCA) (Vector Laboratory), followed by Streptavidin-PE (eBiosciences) and anti-PE microbeads (Miltenyi Biotec). The cell suspensions were loaded onto MACS columns and the pass-through cells were subcloned in 96-well plates to obtain clonal lines. Flow cytometry was used to confirm positive binding to the WT and negative binding to *Fut8^–/–^* hybridoma lines by LCA lectin. All the hybridoma lines were cultured in DMEM with 10% FBS, and supernatants were loaded onto Protein L columns (GenScript) to purify the antibodies. After washing the column with PBS, the bound antibodies were eluted with 0.1 M glycine-HCl (pH 2.5), followed by neutralization with 1.5 M Tris-HCl (pH 8.8). The concentrations of the antibodies were measured by absorbance at OD_280_. Pooled antibodies were sterilized by filtration through 0.22 μm filters before use.

### Tumor models and immunotherapy.

MC38 or luc-B16/F10 cells (1 × 10^5^ cells in 150 μL culture medium) were injected subcutaneously (s.c.) into the flanks of 6- to 8-week-old mice. Mice were treated (i.p.) with αCD39 or CTRL mAb. Gemcitabine was given by intravenous injection (i.v.) through the tail vein. αPD-L1 mAb was given by i.p. injection. Tumor length (L) and width (W) were measured using a caliper every 2–3 days on each mouse. Tumor volume was determined using the formula L × W × W × 0.52.

### IHC.

Paraffin-embedded or frozen sections of tissues were analyzed by IHC as previously described (73). Necrosis was determined with a TUNEL assay kit (Abcam, ab206386). Antibodies used for IHC staining are listed in Supplemental Table 2.

### Flow cytometry.

Single-cell suspensions were prepared from tumors and spleens upon sacrificing the animals. Briefly, tumor tissues (<1 g) were cut into small pieces and digested with a Tumor Dissociation Kit (Miltenyi Biotec) according to the manufacturer’s instructions. Then, cells were filtered through 70 μm strainers, washed with cell staining buffer (BioLegend), and stained with antibodies for flow cytometry. Spleens were also dissected, minced, and filtered through 70 μm strainers. After red blood cell lysis (Lysing Buffer, BD Biosciences), cells were filtered once more through 70 μm strainers, washed, and stained with antibodies for flow cytometry. Antibodies for FACS staining are listed in Supplemental Table 2. FACS data were analyzed using FCS Express 7 software (TreeStar, Inc.).

### ADCC assay with NFAT luciferase reporter Jurkat cells.

Adherent target cells (CHO-mCD39) were seeded in a 96-well plate (8 × 10^3^ cells/100 μL/well) and incubated for 24 hours. Cells were then washed twice with ADCC assay buffer (DMEM or RPMI 1640 medium supplemented with 4% Ultra-Low IgG serum (Thermo Fisher Scientific) and incubated with serially diluted mAbs (CTRL-m1, GoInVivo Purified Mouse IgG1κ control antibody, BioLegend), WT 5F2-m1, WT 5F2-m2c, and Afuc 5F2-m2c αmCD39 antibodies) for 30 minutes at 37°C. Effector cells (NFAT-luc^+^ mFcγRIV Jurkat cells, 3 × 10^6^ cells/mL) were then added to the wells and the mixture (E:T = 1:6) was incubated for 6 hours at 37°C. Bio-Glo Luciferase Assay Reagent (Promega) was finally added into wells and luminescence values were read at 30 minutes using a Synergy Neo2 Multi-Mode Reader (BioTeK Instruments Inc.). ADCC activity was indicated by an increase in luciferase activity over the background.

### Assays for ectonucleotidase activities.

Ectonucleotidase assays were performed using peritoneal mononuclear cells from WT (and mutant) mice. These cells were seeded in a 96-well plate (2 × 10^5^ cells/200 μL/well), incubated for 3 hours, and washed twice to remove unattached cells. Then, fresh medium was added and cells were incubated in 100 μL of medium with antibodies (CTRL-m1, WT 5F2-m1, WT 5F2-m2c, and Afuc 5F2-m2c αmCD39 antibodies) for 24 hours or 26 μM POM-1 (TOCRIS) for 30 minutes. For ectonucleotidase analyses, 100 μL of 100 μM ATP (Sigma-Aldrich) was added to each well and incubated for 20 minutes. Supernatants (25 μL) were transferred into a 96-well opaque-walled multiwell plate (BrandTech Scientific) and mixed with 50 μL of CellTiter-Glo Reagent (Promega) for 2 minutes. Samples were incubated for 10 minutes at room temperature in the dark and then luminescence was read using a Synergy Neo2 Multi-Mode Reader.

### Trogocytosis.

Mouse monocyte-macrophage cells (J774A.1, acceptor cells, 2 × 10^5^ cells/well) and CHO-mCD39-EGFP (target cells, 5 × 10^4^ cells/well) were cocultured (A:T = 4:1) in a 6-well plate for 24 hours. Then, cells were exposed to different concentrations of αCD39 mAb for 48 hours. The levels of CD39 on both acceptor and target cells were analyzed by FACS. After staining samples with αmCD45-BV510, cell apoptosis was assayed with an APC Annexin V Apoptosis Detection Kit and 7-AAD (BioLegend), following the manufacturer’s instructions. For Fc blocking experiments, acceptor and target cells were preincubated with 10 μg/mL FcBlocker or αCD16/αCD32 or its corresponding isotype controls for 30 minutes at 37°C, followed by incubation with αCD39 mAb for 48 hours. EGFP signal on CHO-mCD39-EGFP cells was quantified by FACS.

### Live cell imaging.

J774A.1 (8 × 10^4^ cells/well) and CHO-mCD39-EGFP (2 × 10^4^ cells/well) were seeded into 4-well Chambered Coverglass (Nunc Lab-Tek, 155383, Thermo Fisher Scientific) and incubated in a cell culture incubator for 24 hours. Then, a control antibody or αCD39 mAb was added to the coculture media. The final concentration of mAbs was 2 μg/mL. Thirty minutes later, time-lapse images were acquired with a Zeiss LSM 880 laser-scanning microscope, using a Plan-Apochromat 20×/0.8 M27 objective lens. Cells were imaged on chamber slides suitable for confocal microscopy using an argon 488 nm laser for the EGFP signal, while the differential interference contrast (DIC) channel was used to image nonfluorescent cells. Images were acquired for multiple positions at 2-minute intervals for a total period of 250 minutes (over 4 hours) and later compiled into a time-lapse video for analysis.

### Fluorescent antibody biodistribution.

Afuc αCD39 mIgG2c was first labeled with the AF750 using SAIVITM Rapid Antibody Labeling Kits (Thermo Fisher Scientific, S30046). Then, 5 mg/kg AF750-labeled αCD39 mAb was administered into MC38 tumor–bearing mice on day 11 after tumor implantation. Twenty-four hours later, mice were euthanized, and tumors and vital organs were harvested. One hour before euthanizing, Hoechst 33342 (Thermo Fisher Scientific, H3570) was administered via the tail vein at 15 mg/kg to stain the nuclei of cells and show the functional vasculature in the tumor. Tissues were flash-frozen in a tissue-freezing medium using isopentane chilled on dry ice and cut for histology on a cryostat (10-μm slices). Slides were scanned by a microscope scanner (PANNORAMIC MIDI II, 3DHISTECH).

### Statistics.

Statistical analyses were performed using GraphPad Prism 8. The statistical analysis methods are listed in the figure legends. Significance was defined as a *P* value of less than 0.05. The error bars in the figures represent the standard error of the mean (SEM) or the SD.

### Study approval.

All animal experiments were approved by the IACUC of Beth Israel Deaconess Medical Center (protocol 073-2021).

## Author contributions

SCR, H Zhang, LF, and WG conceptualized the study. H Zhang, LF, KE, and WG developed the methodology. H Zhang, LF, PAM, CM, RN, EC, and LLYC carried out the investigation. H Zhang wrote the original draft of the manuscript. LF, PAM, CM, RN, EC, LLYC, H Zhao, KE, WG, and SCR reviewed and edited the manuscript. H Zhang generated figures. SCR and LF acquired funding. SCR, and WG provided resources. H Zhao and SCR supervised the study. The order of co–first authors was assigned according to the volume of work each contributed to the study.

## Supplementary Material

Supplemental data

Supplemental video 1

## Version 1 07/01/2022

Electronic publication
