# Supplementary material for: Glycoengineered anti-CD39 promotes anticancer responses by depleting suppressive cells and inhibiting angiogenesis in tumor models
Source: J Clin Invest. 2022 Jul 1;132(13):e157431. doi: 10.1172/JCI157431 (PMC9246388; doi:10.1172/JCI157431)
Supplement: Supplemental data [file jci-132-157431-s151.pdf]

Supplemental figures:

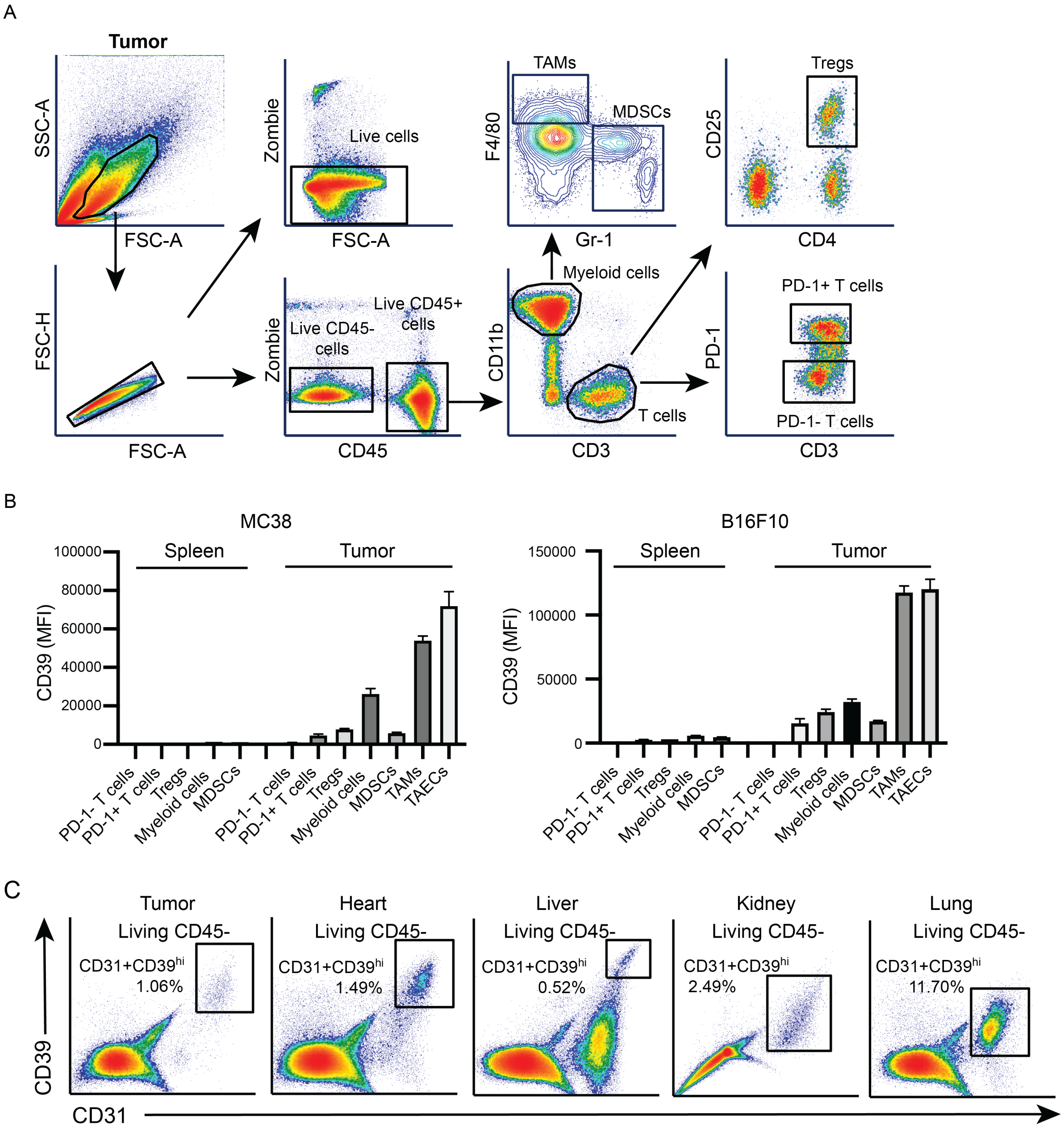

Supplemental figure 1. CD39 expression on tumor-associated endothelial cells (TAECs) and tumor-associated macrophages (TAMs).

1×10^5 MC38 or B16F10 tumor cells were injected subcutaneously into WT C57BL/6 mice. Tissues were harvested on Day 11 (MC38) and Day 14 (B16F10) post tumor cells inoculation.

(A) Gating strategy of FACS analysis.

(B) Quantification of CD39 median fluorescence intensity (MFI) on different cell subsets in tumor and spleen.

(C) Representative FACS plots showing CD39 MFI on endothelial cells of different organs from MC38 tumor-bearing mice.

Data are representative of at least two independent experiments (B-C).

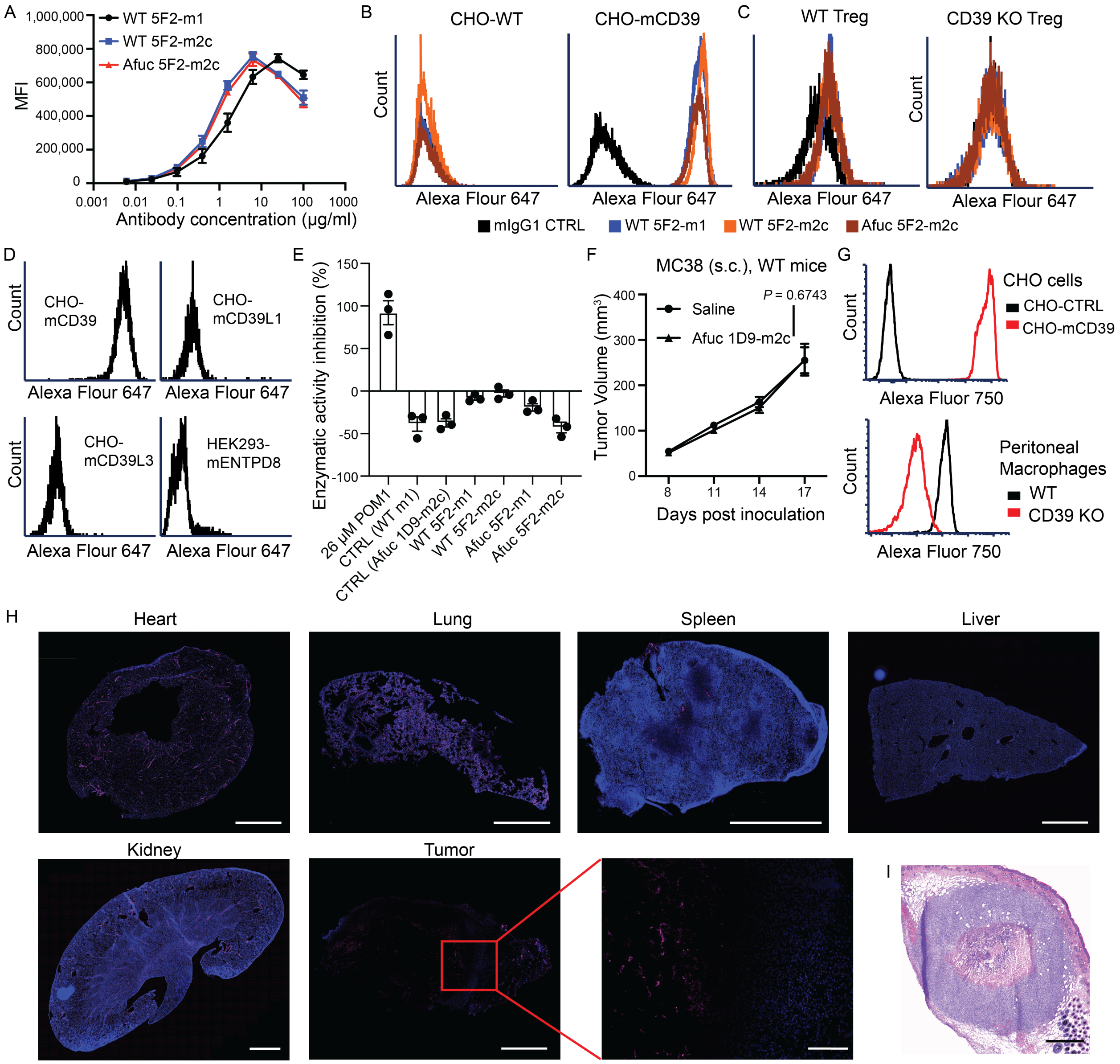

Supplemental figure 2. Antibody isotype-switching and genetic engineering of the hybridomas does not alter the binding affinity and specificity of the antibodies.

(A) Binding of indicated mouse anti-mCD39 antibody (clone 5F2) Fc variants on CHO-mCD39 was assessed by FACS analysis. Data are represented as mean ± s.d..

(B-C) Binding of different anti-mCD39 antibody (clone 5F2) Fc variants to CHO wild type (WT), CHO expressing mouse CD39 cells and Treg cells from the spleen of WT or CD39 KO mice assessed by FACS analysis.

(D) Binding of Afuc 5F2-m2c to CHO cells expressing different members of the ectonucleoside triphosphate diphosphohydrolase family, as assessed by FACS analysis.

(E) Impact of different 5F2 antibody Fc variants on CD39 activity of mouse peritoneal macrophages assessed by luminescent assay.

(F) $1x10^5$ MC38 cells were inoculated subcutaneously into the right flank of the mice (Day 0). MC38-bearing mice received treatment of saline or 5 mg/kg of Ab on Days 8, 11, 14, and 17. Saline (n = 5), Afuc 1D9-m2c (n = 5). Tumor growth was assessed.

Data were shown as mean ± s.e.m. (E-F); Two-way ANOVA was used for statistical analysis (F).

(G) Binding of Alexa-Fluor 750 labeled αCD39 mAb to CHO wild type (WT), CHO expressing mouse CD39 cells and peritoneal macrophages from WT or CD39 KO mice assessed by FACS analysis.

(H) Large field of views of tumor and other organs in Figure 2H. Scale bar: 1000 μm (large views of tissues), and 200 μM (magnified view of tumor).

(I) H&E staining of tumor in Figure 2H. Scale bar: 500 μm.

Data are representative of at least two independent experiments (A-I).

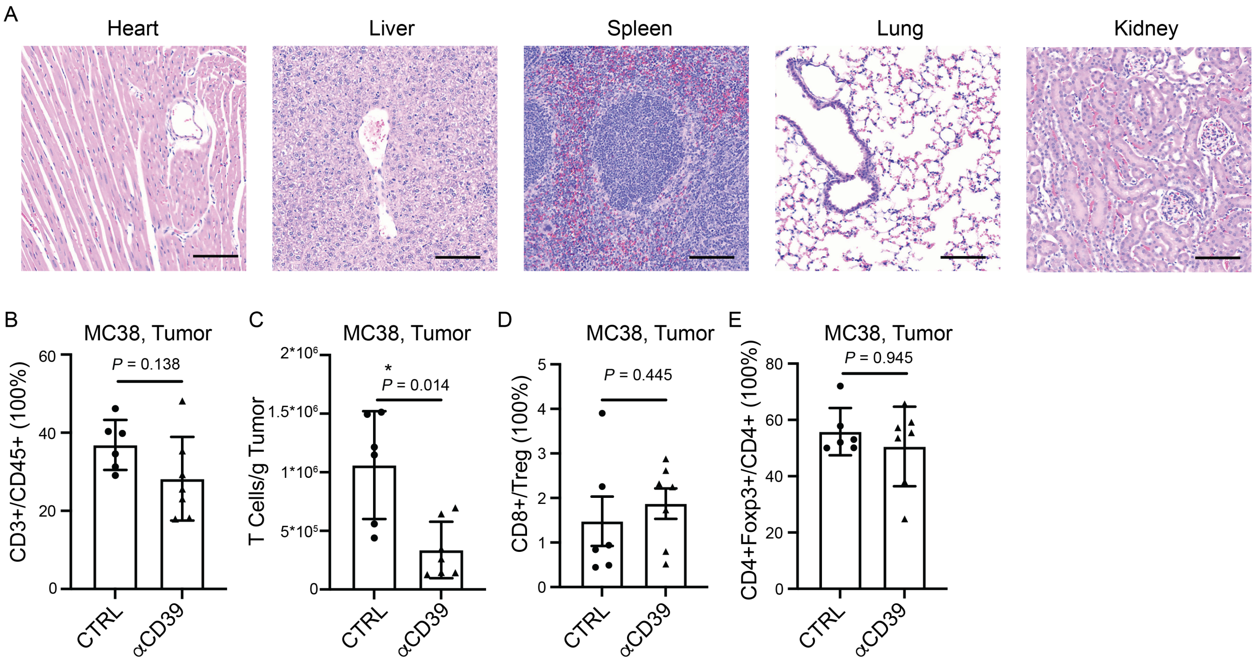

Supplemental figure 3. αCD39 mAb induced cell depletion is selective.

(A) Two doses (200 μg per mouse) of αCD39 mAb were injected intraperitoneally every two days into five healthy C57BL/6 WT mice. Tissues were harvested one day after the second dose of treatment and subjected to IHC analysis. Representative IHC images of the heart, liver, spleen, lung, and kidney of treated mice (n = 5) are shown. Scale bar: 100 μm.

(B-E) 1×10^5 MC38 tumor cells were injected subcutaneously into Foxp3EGFP knock in reporter mice (Day 0). Tumor tissues were collected on Day 12 after treatment with 5 mg/kg of Ab on Days 8 and 11. Quantification of (B) T cells percentage in the tumor-infiltrating immune cells and (C) density of T cells in the tumor. Quantification of the cell ratio of CD8/Treg (D) and Treg/Teff (E) within the tumor.

Data are representative of at least two independent experiments (A-E).

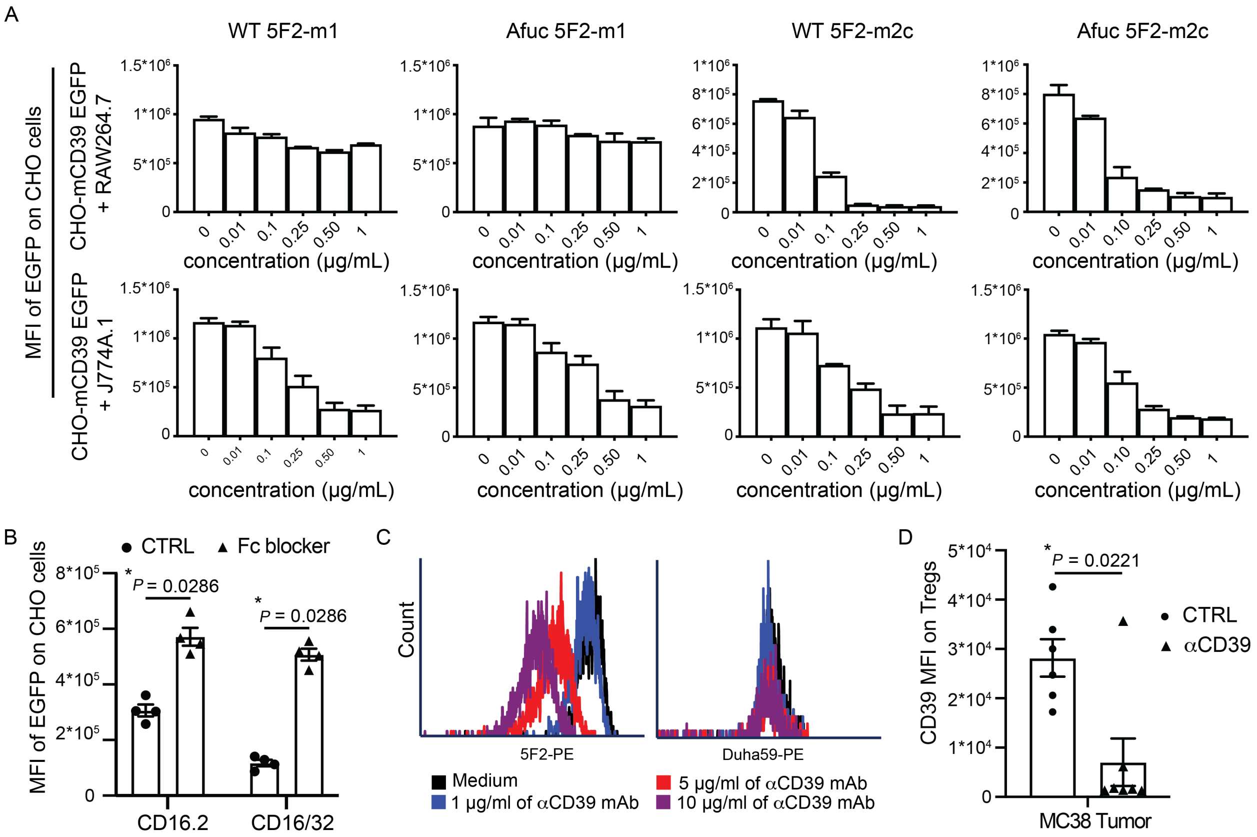

Supplemental figure 4. Effector cells and FcγRs involved in αCD39 mAb induced trogocytosis.

(A) CHO-mCD39-EGFP cells were cultured with either RAW264.7 cells or J774A.1 cells. The co-cultures were then treated with different mAbs concentrations as indicated. EGFP signal on CHO-mCD39-EGFP cells was quantified by FACS.

(B) Co-cultures of J774A.1 and CHO-mCD39-EGFP were pretreated with 10 μg/mL of FcBlocker™ (CD16.2 or CD16/32) or its corresponding isotype controls for 30 minutes and then incubated with αCD39 mAb for 48 hours. EGFP signal on CHO-mCD39-EGFP cells was quantified by FACS. Mann-Whitney test was used for statistical analysis. Data are shown as mean ± s.e.m.; pooled data from 4 independent experiments.

(C) CHO cells overexpressing mouse CD39 (CHO-mCD39) were incubated with the indicated amount of αCD39 mAb for 30 minutes and then were stained with different clones of fluorescence-conjugated anti-CD39 mAb. FACS analysis of the fluorescence on stained CHO-mCD39 cells.

(D) MC38 tumor-bearing mice were treated with 2 doses of 5 mg/kg of CTRL or αCD39 mAb on Days 8 and 11 post tumor implantation. Tumor tissues were harvested 24 hours after the second dose of treatment and processed for FACS analysis. Data are shown as mean ± s.e.m.; Mann-Whitney testing was used for the statistical analysis.

Data are representative of at least two independent experiments (A-D).

Supplemental Table 1: Liver and renal toxicity studies of αCD39 mAb in healthy tumor-free mice

Sample name	ALT(28-132mg/dl)	AST(59-247mg/dl)	BUN (18-29mg/dl)
CTX1	33	29	20
CTX2	0	27	18
CTX3	42	29	16
CTX4	17	31	34
CTX5	27	27	13
CTX6	26	25	12
CTX7	23	21	16
CTX8	15	27	14
CTX9	13	14	15
CTX10	24	17	11

Supplemental Table 1. Three doses of αCD39 mAb at 100 µg (CTX5-7) or 1000 µg (CTX8-10) were injected intraperitoneally into healthy C57BL/6 WT mice on days 1, 4, and 7. Mice received 200 µl of saline (CTX1) or 100 µg of IgG2c isotype control antibody (CTX2-4) served as controls. Plasma samples of mice were harvested one day after the third dose of treatment and subjected to the measurement of ALT, AST and BUN concentrations.

Supplemental Table 2: Antibody reagents

**Reagents**	**Company**	**Cat#**
FITC anti-mouse CD45 Antibody	BioLegend	103108
Brilliant Violet 510™ anti-mouse CD45 Antibody	BioLegend	103138
PE/Cyanine5 anti-mouse CD3ε Antibody	BioLegend	100310
Brilliant Violet 421™ anti-mouse CD3 Antibody	BioLegend	100228
Alexa Fluor® 700 anti-mouse CD4 Antibody	BioLegend	100536
APC/Fire™ 750 anti-mouse CD4 Antibody	BioLegend	100568
Brilliant Violet 510™ anti-mouse CD4 Antibody	BioLegend	100559
APC/Fire™ 750 anti-mouse CD4 Antibody	BioLegend	100568
FITC anti-mouse CD4 Antibody	BioLegend	100510
Brilliant Violet 711™ anti-mouse CD8a Antibody	BioLegend	100759
Brilliant Violet 605™ anti-mouse CD8a Antibody	BioLegend	100744
PE anti-mouse TIGIT (Vstm3) Antibody	BioLegend	142104
Brilliant Violet 785™ anti-mouse NK-1.1 Antibody	BioLegend	108749
Brilliant Violet 421™ anti-mouse CD279 (PD-1) Antibody	BioLegend	135221
APC anti-mouse CD279 (PD-1) Antibody	BioLegend	135210
Alexa Fluor® 700 anti-mouse/human CD11b Antibody	BioLegend	101222
PerCP/Cyanine5.5 anti-mouse/human CD11b Antibody	BioLegend	101228
BV480 Rat Anti-Mouse Ly-6G and Ly-6C	BD Bioscience	746614
FITC anti-mouse Ly-6G/Ly-6C (Gr-1) Antibody	BioLegend	108406
APC anti-mouse CD39 Antibody	BioLegend	143810
PE/Cyanine7 anti-mouse CD39 Antibody	BioLegend	143806
PE anti-mouse CD39 Antibody	BioLegend	143804
PE/Cyanine7 anti-mouse F4/80 Antibody	BioLegend	123114
APC anti-mouse F4/80 Antibody	BioLegend	123116
F4/80 Antibody, anti-mouse, PerCP-Vio® 700, REAfinity™	Miltenyi Biotec	130-118-327
Brilliant Violet 605™ anti-mouse CD274 (B7-H1, PD-L1) Antibody	BioLegend	124321
PE/Cyanine7 anti-mouse CD274 (B7-H1, PD-L1) Antibody	BioLegend	124314
PE anti-mouse CD25 Antibody	BioLegend	101904
Alexa Fluor® 488 anti-mouse CD25 Antibody	BioLegend	102017
Brilliant Violet 605™ anti-mouse CD31 Antibody	BioLegend	102427

Rat anti-mouse CD31 antibody	BD Bioscience	550274
Rabbit anti-mouse CD39 polyclonal Abs	Dr. Jean Sévigny ,Laval University, Canada	
Anti- mouse PD-L1antibody	BioLegend	124329
Anti-mouse CD16/CD32 antibody	Bioxcell	BE0307
Anti-mouse CD16.2 antibody	BioLegend	149502
Zombie NIR™ Fixable Viability Kit	BioLegend	423106

Supplemental Video 1: Macrophages engulf CD39 on the target cells mAb induced trogocytosis

CHO-mCD39-EGFP cells were cultured with J774A.1 cells. Live-cell imaging showing J774A.1 cells in the co-cultures that were treated with αCD39 mAb remove CD39-EGFP from CHO cells and then engulf this transferred CD39-EGFP.
